# Bidirectional GPR119 Agonism Requires Peptide YY and Glucose for Activity in Mouse and Human Colon Mucosa

**DOI:** 10.1210/en.2017-03172

**Published:** 2018-02-19

**Authors:** Iain R Tough, Sarah Forbes, Herbert Herzog, Robert M Jones, Thue W Schwartz, Helen M Cox

**Affiliations:** 1 King’s College London, Wolfson Centre for Age-Related Diseases, Institute of Psychiatry, Psychology & Neuroscience, London, United Kingdom; 2 Garvan Institute of Medical Research, Darlinghurst New South Wales, Sydney, Australia; 3 Department of Medicinal Chemistry, Arena Pharmaceuticals, San Diego, California; 4 Section for Metabolic Receptology and Enteroendocrinology, Novo Nordisk Foundation Centre for Basic Metabolic Research, University of Copenhagen, Copenhagen, Denmark

## Abstract

The lipid sensor G protein–coupled receptor 119 (GPR119) is highly expressed by enteroendocrine L-cells and pancreatic *β*-cells that release the hormones, peptide YY (PYY) and glucagonlike peptide 1, and insulin, respectively. Endogenous oleoylethanolamide (OEA) and the dietary metabolite, 2-monoacylglycerol (2-OG), can each activate GPR119. Here, we compared mucosal responses with selective, synthetic GPR119 agonists (AR440006 and AR231453) and the lipids, OEA, 2-OG, and *N*-oleoyldopamine (OLDA), monitoring epithelial ion transport as a readout for L-cell activity in native mouse and human gastrointestinal (GI) mucosae. We also assessed GPR119 modulation of colonic motility in wild-type (WT), GPR119-deficient (GPR119^−/−^), and PYY-deficient (PYY^−/−^) mice. The water-soluble GPR119 agonist, AR440006 (that cannot traverse epithelial tight junctions), elicited responses, when added apically or basolaterally in mouse and human colonic mucosae. In both species, GPR119 responses were PYY, Y1 receptor mediated, and glucose dependent. AR440006 efficacy matched the GI distribution of L-cells in WT tissues but was absent from GPR119^−/−^ tissue. OEA and 2-OG responses were significantly reduced in the GPR119^−/−^ colon, but OLDA responses were unchanged. Alternative L-cell activation via free fatty acid receptors 1, 3, and 4 and the G protein–coupled bile acid receptor TGR5 or by the melanocortin 4 receptor, was unchanged in GPR119^−/−^ tissues. The GPR119 agonist slowed transit in WT but not the PYY^−/−^ colon *in vitro*. AR440006 (intraperitoneally) slowed WT colonic and upper-GI transit significantly *in vivo*. These data indicate that luminal or blood-borne GPR119 agonism can stimulate L-cell PYY release with paracrine consequences and slower motility. We suggest that this glucose-dependent L-cell response to a gut-restricted GPR119 stimulus has potential therapeutic advantage in modulating insulinotropic signaling with reduced risk of hypoglycemia.

## Teaser

Changes in epithelial function were measured in mouse and human mucosa. GPR119 signaling required endogenous PYY and glucose and was stimulated by apical or basal stimuli and slowed motility.

G protein–coupled receptor (GPCR) 119 (GPR119) is a lipid metabolite sensor that exhibits affinity for the endogenous anorectic lipid, oleoylethanolamide (OEA), as well as metabolites of dietary triglycerides, such as 2-monoacylglycerol (2-OG) (1). The receptor is highly expressed by pancreatic *β*-cells and gastrointestinal (GI) enteroendocrine K-cells and L-cells (2–6). This relatively restricted expression pattern translates potentially to reduced risk of receptor-mediated side-effects; however, low-level GPR119 expression in skeletal muscle may perturb fatty acid metabolism, leading to dyslipidemia (7). Cellular GPR119 G*α*s signaling raises intracellular cyclic adenosine monophosphate levels, and in *β*-cells, this leads to glucose-stimulated insulin release (2) and in L- or K-cells, to hormone secretion, *i.e.*, glucagonlike peptide 1 (GLP-1), peptide YY (PYY), and glucose-dependent insulinotropic peptide (GIP) (3, 8–10). Additionally, the glucose dependence of GPR119-triggered incretin release reduces the possibility of hypoglycemia (3, 8–11). The maximization of GPR119 activity through selective agonism, in combination with either dipeptidyl peptidase 4 inhibition to stabilize released hormones or with long-chain free fatty acid (LCFA) activators of free fatty acid receptor 1 (FFA1) (12), offers improved potential treatment of type 2 diabetes (1, 6, 13, 14). However, to minimize detrimental muscular effects (7) a gut-restricted approach would be preferential, assuming that GPR119 is located on luminal L-cell (and K-cell) membranes. Whether GPR119 is trafficked to luminal [apical (ap)] or blood-borne (basal) surfaces (or both) is unclear currently. Furthermore, the GPR119 agonists available, to date, are lipophilic and often lacking GPR119 selectivity (11). This and other nutrient-sensing GPCRs may be differentially targeted, and we have used native mucosal preparations that retain mucosal polarity to determine the sidedness of responses to, for example, acyl lipid mimetics, LCFA-stimulated FFA1 and free fatty acid receptor 4 (FFA4) signaling, or L-amino acids that act via the calcium-sensing receptor (8, 10, 15, 16). Each of these nutrient mechanisms can be stimulated apically or basolaterally, but the commercially available ligands specific for GPR119, FFA1, or FFA4 are lipid soluble, so are unlikely to be restricted to one surface. A primary aim of this study was therefore to determine the sidedness of GPR119 responses in murine and human GI mucosae using a water-soluble agonist, AR440006.

Previously, the “first-in-class” GPR119 agonist PSN632408 was used to characterize L-cell activation via endogenous PYY release and stimulation, predominantly of epithelial Y1 receptors. PSN632408 improved glucose tolerance in wild-type (WT) mice, but this was compromised in PYY-deficient (PYY^−/−^) mice (8). Subsequently, a more potent, selective GPR119 agonist (PSN-GPR119) was used to cause PYY, GLP-1, and GIP secretion, and crucially, the GPR119 agonist retained glucose-dependent efficacy in diabetic rat and mouse GI mucosae (10). Interestingly, this agonist exhibited reduced efficacy compared with PSN632408 (8), raising the possibility of GPR119 desensitization with more potent ligands with incretin relevance. GPR119 also exhibits high constitutive activity in transfected cells (2, 17, 18), but the degree to which this occurs in native tissue has not yet been established. A selective antagonist (AR436352) inhibits GPR119 constitutive activity and GPR119 agonism (17). Small molecule GPR119 agonists, with improved selectivity, are also now available and have been shown to stimulate PYY, as well as GLP-1 release from L-cells in culture (19) and in native mucosae (8, 10). The similarity in cellular distribution of PYY-sensitive Y1 and Y2 receptors in the human and mouse colon is worthy of note (20–22), particularly in the context of nutrient-stimulated L-cell activation (23), and thus, resolving these mechanisms in mouse GI tissues has translational impact.

Locally produced lipids, such as OEA, activate GPR119 at micromolar concentrations in recombinant cells (24, 25), and in native tissues, this endogenous activity would be indistinguishable from constitutive GPR119 activity. The metabolism of endogenous OEA, by the membrane fatty acid amide hydrolase (FAAH), implicates the enzyme as an alternative target for amplifying incretin release with antidiabetic potential. The availability of selective FAAH inhibitors (*e.g.*, URB597) (25) thus enables the interrogation of endogenous OEA mechanisms in native tissues, including those from knockout mice [GPR119 deficient (GPR119^−/−^)]. Interestingly, the hypophagic effects of OEA appear to be independent of GPR119 (26), whereas its stimulation of L-cell GLP-1 release (and that of AR231453 and 2-OG) is significantly impaired in GPR119^−/−^ enteric cultures (27). GPR119^−/−^ mice fed a standard diet have been reported to exhibit normal glucose-stimulated insulin release and GLP-1 secretion, but they lack sensitivity to the GPR119 agonist AR231453 (2, 3), including its attenuation of gastric emptying (28). A recent description of a *β*-cell-specific GPR119^−/−^ mouse described normal acute glucoregulatory responses (29), indicating that GPR119 signaling in L-cells is likely to be the primary site of lipid GPR119-mediated sensing. Therefore, we also set out to establish the specificity of GPR119 signaling in native mucosae from the mouse and human GI tract and whether FAAH inhibition would amplify GPR119 signaling in the mouse colon.

Given the growing significance of intestinal GPR119 signaling in glucoregulation, we determined the selectivity of three lipids [OEA, 2-OG, and *N*-oleoyldopamine (OLDA)], comparing their activities with those of AR231453 and a water-soluble synthetic agonist, AR440006, in GI tissues from WT and GPR119^−/−^ mice, including the GPR119 antagonist, AR436352 (17). We show that GPR119 activation on the luminal (ap) or the blood-borne [basolateral (bl)] surfaces is L-cell PYY mediated and that these GPR119-specific responses are glucose dependent in the WT mouse GI tract and in human colonic mucosa.

## Materials and Methods

### Animals

All animal experimentation and care were conducted in compliance with UK Home Office regulations [Animals (Scientific Procedures) Act 1986] and were approved by the local Ethics Committees in King’s College London and the University of Copenhagen (Animal Experimental Inspectorate of the Danish Minister of Justice: Project Number 13.242). WT adult mice (12 weeks or older, of either sex) and the background strain of C57BL/6J (GPR119^+/+^ and GPR119^−/−^) mice or the mixture of C57BL/6-129SvJ (PYY^+/+^ and PYY^−/−^) mice were used as indicated. Mice were housed conventionally in open-top cages under a 12-hour light-dark cycle (0700 to 1900 hours) at 20 to 24°C and 55% (±10%) humidity with free access to standard chow (Rat and Mouse No.3 Breeding diet; Special Diets Services, Braintree, UK) and tap water.

### Measurement of short-circuit current across mouse or human mucosal preparations

Mucosae from different regions of the GI tract of WT, GPR119^−/−^, or PYY^−/−^ mice or normal human colon were prepared, bathed in Krebs-Henseleit (KH) buffer, and voltage clamped at 0 mV in Ussing chambers, as described previously (8, 20, 30). Human colon specimens were obtained from consenting patients undergoing elective bowel resection surgery for cancer of the large bowel (as approved by the Research Ethics Committee of Guy’s and St Thomas’ National Health Service Foundation Trust). Vectorial ion transport was measured as short-circuit current (Isc; microamps divided by centimeters squared), and once initial baseline Isc and transepithelial resistance (TER; +1.0 mV for 5 seconds every 250 seconds) measurements had been made, drugs were added to the ap or bl reservoir, as indicated. GPR119 antagonist pretreatment occurred 15 minutes before addition of the secretagogue, vasoactive intestinal polypeptide (VIP; 10 nM, bl), followed by a single concentration only of a ligand, *e.g.*, GPR119 agonist (ap or bl). These ligand-induced reductions in Isc were measured for 20 minutes before a control PYY (10 nM, bl) addition. Endogenous PYY mediation of GPR119 activity was determined using optimized blocking concentrations of the competitive Y1 antagonist (BIBO3304, 300 nM, bl) and/or Y2 antagonist (BIIE0246, 1 µM, bl) (31). In glucose-sensitivity studies, mouse colonic mucosa was bathed either with KH containing glucose (11.1 mM) on both sides, or adjacent preparations were bathed with KH, in which glucose was replaced by mannitol (11.1 mM) in either the ap or the bl reservoir (8, 10, 16, 30). As a control, blockade of the apically directed sodium/glucose cotransporter 1 (SGLT1) was confirmed using ap phloridzin (50 µM). The electrogenic response to phloridzin (a reduced Isc) was absent when mannitol replaced glucose in the ap reservoir only. Additionally, the effect of phloridzin (50 µM, ap) and phloretin (100 µM, both sides) on GPR119 signaling was tested by pretreating descending colon mucosa with the SGLT1 and glucose transporter 2 inhibitors, followed 15 minutes later by VIP (10 nM, bl), then AR440006 (300 nM, ap), and finally, PYY (10 nM, bl).

In time-course experiments with a maximal concentration of AR440006 (1 µM) or agonists selective for FFA1 (TAK875, 3 µM) (16), FFA receptor 3 (FFA3; AR420626, 100 nM), or FFA4 (Merck A, 10 µM); G protein–coupled bile acid receptor, TGR5 (Merck V, 1 µM) (32) or GP-A (3 µM) (33), each drug was added to either the ap or bl reservoir (10 minutes after 10 nM VIP). The resultant free fatty acid (FFA)/TGR5-induced decreases in Isc were monitored for 20 minutes and data pooled from four different WT (C57BL/6-129SvJ) colonic specimens. Mucosal TER was recorded throughout and TER data presented at the time (t) of agonist addition (t = 0 minute) compared with that at the peak of each agonist response (t = 5 to 10 minutes).

### Liquid chromatography–tandem mass spectrometry

To determine whether AR440006 crossed mucosal preparations during the GPR119 response, bathing fluid was sampled at agonist addition (t = 0 minutes, control) and 10 minutes later when AR440006 responses were maximal. Particulate matter was removed and samples diluted in 0.1% formic acid in 50% methanol for liquid chromatography (LC)–tandem mass spectrometry (MS/MS; Thermo Accela pump and autosampler coupled to a Thermo LTQ XL ion trap mass spectrometer, LC column: Thermo Hypersil Gold aQ column, 50 mm × 2.1 mm × 3 µm; Thermo Fisher Scientific, Waltham, MA). Aliquots (10 µL) were analyzed for 10 minutes (flow rate 200 µL/min) in a gradient of 0.1% formic acid in water (A) and 0.1% formic acid in acetonitrile (B). The initial gradient conditions were 5% B, held for 0.2 minute, then increased to 95% B at 3 minutes. Ninety-five percent B was maintained for 2 minutes, and the column was re-equilibrated at 5% B over 0.5 minute and held for 4.5 minutes (total run time 10 minutes). Mass spectrometry [MS; full MS over mass range mass-to-charge ratio (*m*/*z*) 350 to 550, full MS/MS of *m*/*z* 400 over mass range *m*/*z* 110 to 450 at collision energy 15 Arb] used electrospray with positive polarity, an ionization spray voltage (4000 V), capillary voltage (12 V), temperature (350°C), and sheath gas flow (50 Arb). With the use of this method, AR440006 exhibited a retention time of 4.2 minutes, and product ion *m*/*z* 280 was used for AR440006 determination. The relative abundance was calculated as the area of this peak in samples from mucosa exposed to ap or bl AR440006 (at t = 0 and t = 10 minutes).

### Measurement of basal rates and GPR119 agonist-stimulated colonic transit *in vitro* and *in vivo*

For colonic transit measurement *in vitro*, the entire mouse colon was removed, measured, and photographed to provide the initial position (t = 0 min) of fecal pellets from the cecal junction to the rectum. Individual colons were placed in KH buffer with vehicle (0.1% ethanol) PSN632408 (10 µM) for 20 minutes before a second photograph was taken to establish fecal pellet transit, as described previously (31). For *in vivo* upper-GI and colonic motility measurement, male and female WT mice were acclimatized to handling for 3 days before experimentation and were fasted overnight (for up to 16 hours). Vehicle (100 µL, buffered saline) or AR440006 [30 mg/kg, intraperitoneally (IP)] was administered 10 minutes before rectal-bead insertion, measuring the time to bead excretion as described previously (31, 34). Upper-GI transit was measured following intragastric delivery of a charcoal meal, 15 minutes after IP vehicle or AR440006 administration, recording the distance traveled by the charcoal front in 30 minutes (and quoted as a percentage of the total small intestinal length) (31, 34).

### Statistical analyses

Results are expressed as means ± standard error of the mean (SEM) from numbers of observations, as stated. These data were analyzed using GraphPad Prism (version 6.07; GraphPad Software, La Jolla, CA), and statistical differences were identified using one-way analysis of variance (ANOVA) with Dunnett (or Tukey) *post hoc* test, as appropriate. For single comparisons between tissues from different genotypes or between vehicle and drug *in vivo*, Student unpaired *t* test was used, and in all cases, *P* < 0.05 was considered statistically different.

### Materials

BIBO3304 and BIIE0246 were purchased from Tocris (Bristol, UK), PSN632408 and OEA were from Cayman Chemical (Ann Arbor, MI), and tetrodotoxin and *α*-melanocyte–stimulating hormone (*α*-MSH) were from Abcam Biochemicals (Cambridge, UK). PYY was purchased from Generon (Slough, UK) and VIP from AnaSpec (Freemont, CA). AM1638, AR231453, AR420626, AR435707, AR436352, AR440006, Merck A, TAK875, and Merck V were provided by R.M.J. and T.W.S. All other chemicals were purchased from Sigma-Aldrich (Poole, UK).

## Results

### Basal electrophysiological parameters of different GI mucosae from mice of different genotypes

Mucosal preparations from WT and GPR119^−/−^ mice were compared, specifically their baseline Isc and TER values (Supplemental Table 1). WT-ascending colon Isc levels were higher than those of other GI areas, whereas jejunum and descending colon Isc levels were higher in GPR119^−/−^ compared with corresponding WT tissues. Additionally, TER values were significantly higher in ascending and descending colon mucosae from GPR119^−/−^ vs WT mice.

### Comparison of the lipid and synthetic GPR119 agonism in the WT and GPR119^−/−^ mouse colon

**Figure 1.**
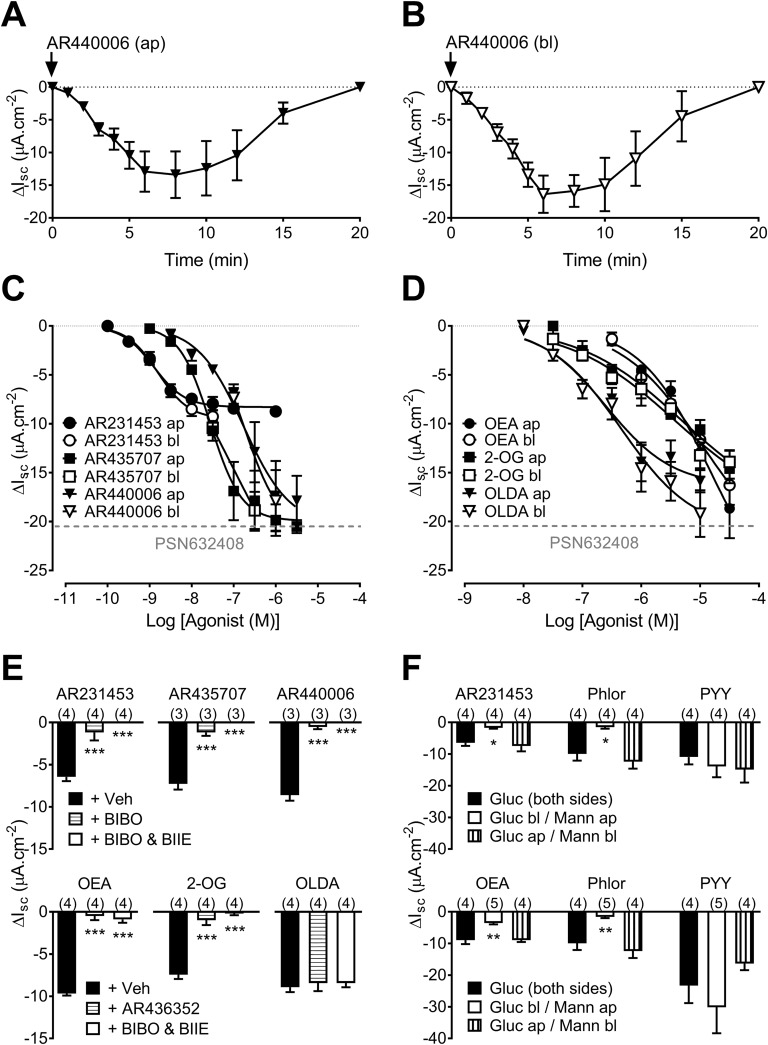
Small molecule and lipid-based GPR119 agonism in mouse-descending colon mucosa. (A) Time dependence of ap and (B) bl AR440006 responses (1 µM, n = 4). (C) Concentration-response curves for three small molecule GPR119 agonists and (D) three lipids added to either the ap or bl reservoirs. The horizontal, dashed, gray lines denote the maximal response to the GPR119 agonist, PSN632408 (ap), published previously (8). (E) Upper histogram shows inhibition of the ap GPR119 agonism (3 nM AR231453, 30 nM AR435707, or 300 nM AR440006) by Y1 and Y2 receptor blockade [300 nM BIBO3304 (BIBO) and 1 µM BIIE0246 (BIIE), respectively] compared with vehicle (Veh; 0.01% dimethyl sulfoxide). In the lower histogram, ap 2-OG (3 µM), OEA (10 µM), or OLDA (300 nM) responses are differentially affected by pretreatment with the GPR119 antagonist, AR436352 (10 µM, ap) or Y antagonists (BIBO and BIIE, as shown previously). (F) AR231453 (10 nM; upper histogram) and OEA (10 µM; lower histogram) responses require the presence of ap glucose (Gluc). In both histograms, phloridzin (Phlor; 50 µM, ap) only reduces Isc when glucose is present apically. Subsequent PYY (10 nM) responses are unaffected by replacement of glucose on either side. Each point or bar is the means ± 1 SEM from n numbers (in parentheses). Statistical differences (ANOVA with Dunnett *post hoc* test) from controls are identified as follows: *P < 0.05, **P < 0.01, and ***P < 0.001. Mann, mannitol; *Δ*I_sc_, change in Isc.

The first-in-class water-soluble agonist AR440006 elicited responses with similar kinetics when added either apically (Fig. 1A) or basolaterally (Fig. 1B), as observed previously with the lipophilic GPR119 agonist PSN632408 (8). LC-MS/MS analysis of ap and bl samples, 10 minutes after addition of AR440006 to either surface, showed that only trace amounts of the drug permeated across the tissue (Supplemental Fig. 1) at a time when the mucosa was responding maximally. The estimated concentration of AR440006 in the opposite compartment, 10 minutes after drug addition, was ∼2 nM, *i.e.*, approximately the threshold concentration for this drug. This indicates that GPR119 receptors are located on both epithelial domains, *i.e.*, facing the lumen (apically) and the lamina propria (basolaterally), and that GPR119 activation by luminal stimuli or lipids, present in the lamina propria, is equally capable of depolarizing L-cells. Next, a comparison of the potency and efficacy of small molecule GPR119 agonists (AR231453 and AR435707) (12), AR440006, and three lipids (OEA, 2-OG, and OLDA) was undertaken in distal colon mucosa, where L-cells predominate. Ap or bl addition of all but one agonist (AR231453) exhibited similar maxima and overlapping response time courses. Interestingly, AR231453 was more potent but appeared to be a partial agonist, its efficacy reaching only 50% of that exhibited by all of the other agonists (Fig. 1C and 1D and Table 1). The half-maximal effective concentration (EC_50_) values for agonists tested apically or basolaterally were similar (Table 1).

**Table 1.** Agonist EC_50_ Values (With Confidence Limits and d.f.) Following Single Additions to Either the ap or bl Side

Agonist (Side Added)	EC_50_	95% Confidence Limits	d.f.	n Numbers
AR231453 (ap)	1.2 nM	0.7–2.2 nM	49	3–16
AR231453 (bl)	1.6 nM	0.9–2.8 nM	21	3–7
AR435707 (ap)	27.7 nM	16.1–47.7 nM	28	3–5
AR435707 (bl)	n.d.	—	—	4
AR440006 (ap)	170.4 nM	37.0–784.5 nM	24	3–4
AR440006 (bl)	n.d.	—	—	4
OLDA (ap)	251.0 nM	97.2–648.4 nM	23	3–4
OLDA (bl)	455.3 nM	121.0 nM–1.7 µM	21	3–4
2-OG (ap)	4.9 µM	184.4 nM–1.2 mM	25	3–6
2-OG (bl)	3.7 µM	159.3 nM–85.2 µM	22	3–4
OEA (ap)	14.7 µM	48.6 nM–4.4 mM	27	3–12
OEA (bl)	10.6 µM	181.6 nM–616.8 µM	19	3–7

Abbreviation: d.f., degrees of freedom; EC_50_, half-maximal concentration; n.d., not determined (as only two bl agonist concentrations were tested and, critically, these responses were identical to their ap counterparts).

GPR119 agonism (with AR compounds) was significantly inhibited by pretreatment with the Y1 antagonist BIBO3304, and the residual GPR119 agonism was abolished by inclusion of the Y2 antagonist (BIIE0246; Fig. 1E, upper histogram). 2-OG responses were also abolished by the combination of Y1 and Y2 blockers, as well as by the GPR119 antagonist AR436352 (Fig. 1E, lower histogram), confirming the GPR119 activity of 2-OG. In contrast, responses to OLDA were unaffected by either Y1/Y2 receptor antagonists or by GPR119 blockade (Fig. 1E, lower histogram), indicating a non-GPR119 mechanism for this lipid’s mucosal response. Importantly, the responses to AR231453 and OEA were glucose sensitive (Fig. 1F), as seen previously for the GPR119 agonism in rodent colon mucosae (8, 10). Internal controls confirmed that replacement of ap glucose with mannitol abolished the ability of the SGLT1 inhibitor, phloridzin, to reduce Isc levels (as SGLT1 is apically located and Na^+^ coupled), whereas PYY responses were predictably glucose insensitive (Fig. 1F). Phloridzin (50 µM, ap) pretreatment also significantly inhibited AR440006 (300 nM, ap) responses (vehicle controls: −8.3 ± 0.7 µA ⋅ cm^−2^; +Phloridzin: −2.0 ± 0.6 µA ⋅ cm^−2^, n = 5, *P* < 0.001). The combination of phloridzin (ap) and phloretin (both sides) abolished AR440006 responses (−0.8 ± 0.5 µA ⋅ cm^−2^, n = 5, *P* < 0.001, compared with control GPR119 responses quoted previously), whereas PYY (and VIP) responses were unaffected by coincident SGLT1 and glucose transporter 2 inhibition (data not shown).

**Figure 2.**
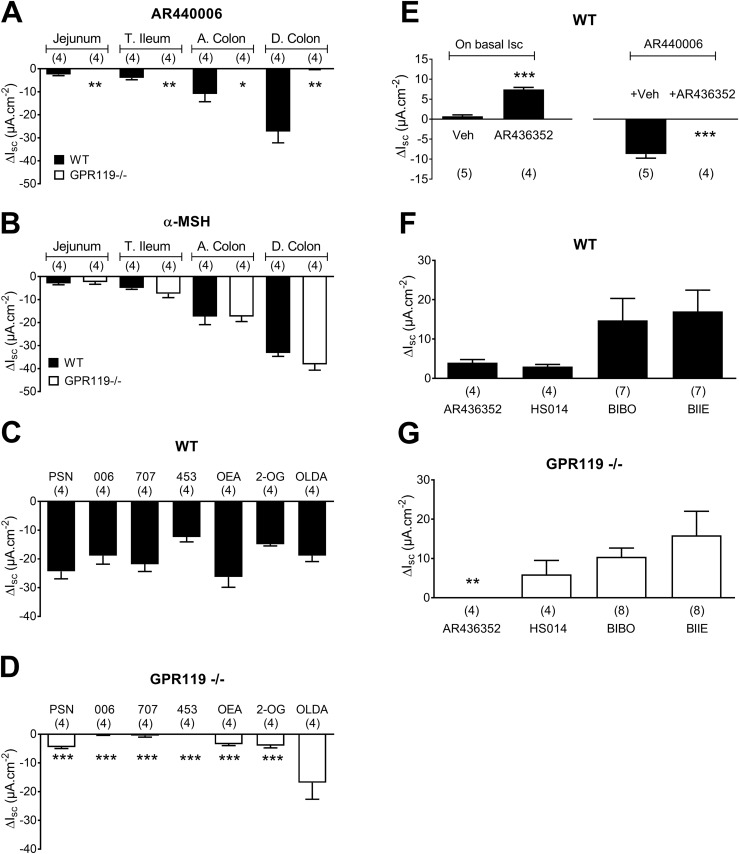
Selectivity of small molecule agonists compared with three lipids in WT and GPR119^−/−^ intestinal and colonic mucosae. (A) Regional variation of responses to the water soluble GPR119 agonist, AR440006 (1 µM; bl), in WT and GPR119^−/−^ jejunum, terminal ileum (T. Ileum), ascending colon (A. colon), and descending colon (D. colon) mucosae. (B) Regional variation of *α*-MSH (1 µM; bl) responses in intestinal mucosae from WT and GPR119^−/−^ mice. Responses to maximally effective concentrations of the small molecule GPR119 agonists, PSN632408 (PSN; 10 µM), AR440006 (006; 1 µM), AR435707 (707; 100 nM), and AR231453 (453; 10 nM), or the lipids, OEA (10 µM), 2-OG (30 µM), and OLDA (30 µM), each added apically in (C) WT or (D) GPR119^−/−^-descending colon mucosa. (E) Endogenous GPR119 tone was revealed by the GPR119 antagonist AR436352 (10 µM; ap) that then abolished subsequent AR440006 (1 µM; ap) responses. (F) GPR119 antagonist AR436352 (1 µM; bl), the MC4 antagonist HS014 (30 nM; bl), Y1 antagonist BIBO3304 (300 nM BIBO; bl), or Y2 antagonist BIIE0246 (1 µM BIIE; bl) revealed tonic activities in WT colonic mucosa, but (G) only GPR119 tone was absent from GPR119^−/−^ mucosa. Values are the means ± 1 SEM with statistical differences as shown: *P < 0.05, **P < 0.01, and ***P < 0.001 (Student *t* test), comparing responses in WT and GPR119^−/−^ tissues.

To establish specific loss-of-GPR119 function and show sidedness of GPR119 receptors, we used GPR119^−/−^ tissues and the water-soluble GPR119 ligand AR440006 (Fig. 2A). Bl *α*-MSH was included (as a control) to activate melanocortin 4 receptors (MC4) that are also expressed by L-cells (30) in four distinct GI areas (jejunum, terminal ileum, ascending, and descending colon; Fig. 2B), and these peptide responses were compared in WT and GPR119^−/−^ mucosae. The bl AR440006 agonism was absent in GPR119^−/−^ tissues, whereas *α*-MSH responses were unchanged in all GPR119^−/−^ tissues (Fig. 2A and 2B). MC4 agonism was confirmed using the MC4 antagonist, HS014 (30 nM), to block all *α*-MSH responses (data not shown), thus indicating normal MC4 G*α*s signaling in L-cells of GPR119^−/−^ mucosa. As seen previously, the size of GPR119 and MC4 responses was greatest in the distal colon (8, 30), following the established pattern of increased L-cell frequency in the distal large bowel. In GPR119^−/−^-descending colon mucosa, all four small molecule GPR119 ligands (namely, PSN632408, AR440006, AR435707, and AR231453) lost considerable activity, as did OEA and 2-OG (Fig. 2C and 2D). In contrast, OLDA responses were a similar size in WT and GPR119^−/−^ tissue (Fig. 2C and 2D), and this lipid response is therefore not GPR119 mediated. OLDA has been suggested as an endogenous transient receptor potential vanilloid 1 (TRPV1) activator (35), but TRPV1 desensitization with capsaicin had no effect on OLDA responses in mouse colon mucosa (data not shown).

The competitive GPR119 antagonist, AR436352 (36), raised basal Isc levels (Fig. 2E), revealing GPR119 tonic activity in WT-descending colon mucosa. Subsequent ap AR440006 responses were abolished (Fig. 2E). OEA responses were also inhibited significantly by the GPR119 antagonist [OEA (10 µM) + antagonist: −5.0 ± 1.0 μA/cm^2^ (n = 4) compared with OEA controls shown in Fig. 2C (n = 4), *P* < 0.001]. GPR119 tone was absent in GPR119^−/−^ tissue (Fig. 2F and 2G). Next, other GPCR tonic activities were compared in the WT and GPR119^−/−^ colon using proven competitive antagonists selective for the MC4, Y1, or Y2 receptors. The MC4 antagonist HS014 revealed tonic MC4 activity (as seen previously) (30) that was similar in the WT and GPR119^−/−^ colon (Fig. 2F and 2G). Y1 and Y2 tonic activities, revealed by using a combination of the competitive Y1 antagonist BIBO3304 and Y2 antagonist BIIE0246, were also unchanged in GPR119^−/−^ mucosa (Fig. 2F and 2G), indicating normal L-cell and endogenous PYY functions in GPR119^−/−^ mucosa.

### FAAH inhibition enhances GPR119 tone and Y1 tone but not MC4 tone in colonic mucosa

**Figure 3.**
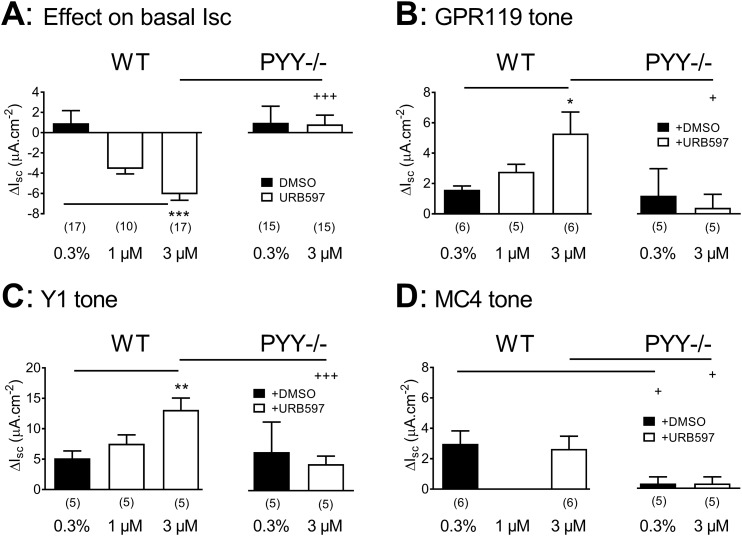
FAAH inhibition with URB597 amplifies GPR119 and Y1 tones but not MC4 tone in the WT- or PYY^−/−^-descending colon. (A) The effects of vehicle [0.3% dimethyl sulfoxide (DMSO)] or URB597 (at 1 or 3 µM, ap) on basal Isc levels in the WT colon (triplet of bars to left) and PYY^−/−^ colon mucosa (right two bars). (B) Pretreatment with URB597 significantly amplifies (at 3 µM) GPR119 tone (revealed using GPR119 antagonist AR436352, 10 µM, ap) in WT (left) but not PYY^−/−^ tissue (right). (C) Y1 receptor signaling (after Y1 antagonist BIBO3304, 300 nM, bl) is increased by URB597 but only in WT mucosa. (D) MC4 tone (revealed using MC4 antagonist HS014, 30 nM, bl) is unaffected by URB597 (3 µM, ap), and MC4 tone is absent in PYY^−/−^ tissue. Values are the means ± 1 SEM, and statistically significant differences from vehicle controls (0.3% DMSO) were identified using ANOVA (with Dunnett *post hoc* test) and shown as: *P < 0.05, **P < 0.01, and ***P < 0.001, whereas differences in tonic activities between WT and PYY^−/−^ are shown as: ^+^*P* < 0.05 and ^+++^*P* < 0.001.

The FAAH inhibitor URB597 has been shown to elevate OEA levels and amplify GPR119 signaling (25), so it was important to establish whether FAAH inhibition in mucosa resulted in enhanced GPR119 tonic activity. First, bl addition of URB597 (3 µM) significantly reduced basal Isc levels in WT colon mucosa, and this effect was absent in PYY^−/−^ tissue (Fig. 3A). Next, FAAH inhibition amplified GPR119 tone in the WT colon (with 3 µM URB597; Fig. 3B, left), and this tonic activity was absent in the PYY^−/−^ colon (Fig. 3B, right). URB597 also amplified Y1 tone in the WT tissue, but it failed to do so in PYY^−/−^ colon mucosa (Fig. 3C). In contrast, URB597 pretreatment had no effect on MC4 tone, revealed by the MC4 antagonist HS-014 (Fig. 3D), but this tonic activity was also absent in PYY^−/−^, as observed previously (30). Thus, FAAH blockade amplified GPR119 and Y1 tonic activities, implicating the potential involvement of endogenous OEA in GPR119- and PYY-induced Y1 tone, but not in MC4 tone. Notably, all three tonic mucosal activities required the presence of endogenous PYY.

### FFA1, FFA3, FFA4, and TGR5 signaling is similar in the WT and GPR119^−/−^ colon

**Figure 4.**
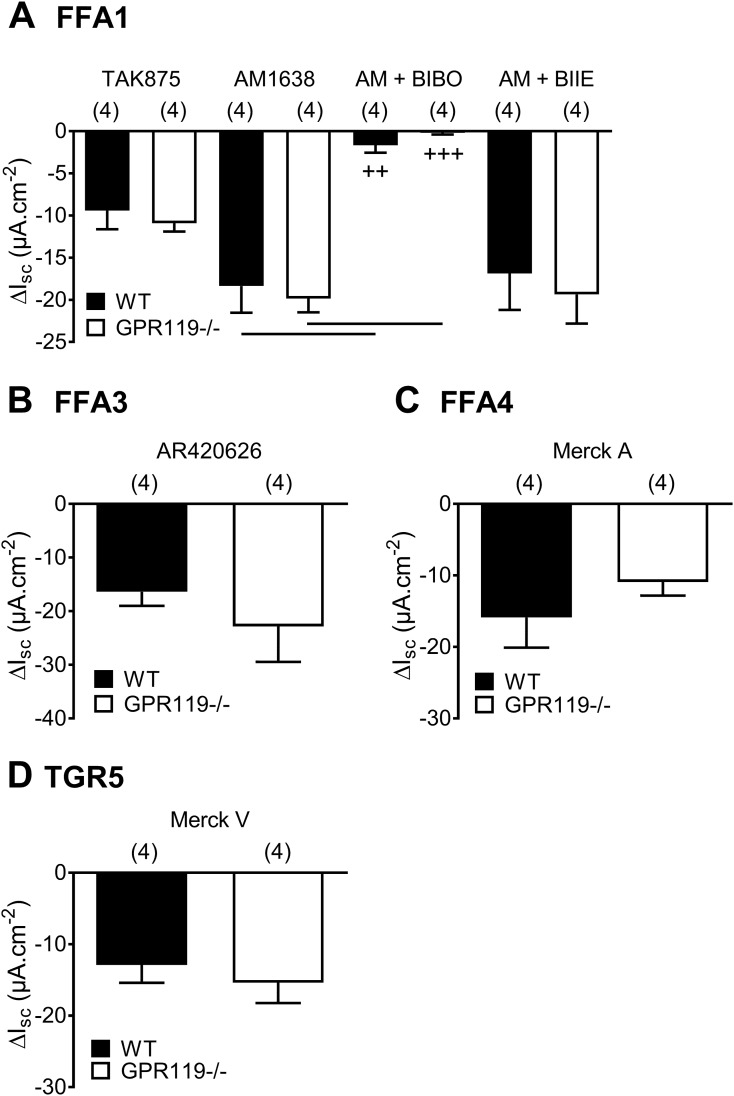
Comparison of selective FFA1, FFA3, FFA4, and TGR5 responses in GPR119^−/−^- and WT-descending colon mucosa. (A) The selective FFA1 agonism with TAK875 (3 µM, ap) or AM1638 (300 nM, ap) ± Y1 antagonist BIBO3304 (300 nM; AM + BIBO) or ± Y2 antagonist BIIE0246 (1 µM; AM + BIIE). (B) The FFA3 agonism (with AR420626, 100 nM), (C) the FFA4 agonism (Merck A, 10 µM), and (D) the TGR5 agonist (Merck V, 1 µM) were each added apically. Values are the means − 1 SEM with statistical differences as shown: **^++^***P* < 0.01 and **^+++^***P* < 0.001, comparing AM1638 responses ± BIBO in WT or GPR119^−/−^ tissues (ANOVA with Dunnett *post hoc* test).

A number of FFA and bile acid GPCRs are expressed by L-cells rather than the surrounding epithelia (37). We set out to establish whether FFA receptor-induced L-cell activation, specifically via FFA1, FFA3, or FFA4, or the bile acid receptor TGR5 were inhibited in GPR119^−/−^ colon mucosa, using proven selective agonists. Single ap agonist additions (using predetermined maximal concentrations that were comparable with equivalent bl responses; Supplemental Fig. 2) each reduced Isc levels, reminiscent of endogenous PYY, and none of these ap responses were significantly altered in GPR119^−/−^ tissue (Fig. 4). Two different FFA1 agonists were tested: the prototypic TAK875 and AM1638, which was included, as this FFA1 signaling occurs via coincident G*α*q and G*α*s (32). In WT and GPR119^−/−^ tissue, AM1638 responses were similar in size and were blocked by the Y1 antagonist (BIBO3304), rather than being Y2 mediated, as pretreatment with the Y2 antagonist BIIE0246 had no substantial effect on AM1638 responses (Fig. 4A). FFA3 and FFA4 agonism (using AR420626 and Merck A, respectively) and the TGR5 agonism using Merck V (Fig. 4D) (32) or GP-A (in the WT colon; Supplemental Fig. 2) (33) were also similar in the WT and GPR119^−/−^ colon (Fig. 4B–4D), showing apparently normal L-cell G*α*q and G*α*s signaling in GPR119^−/−^ mucosa. In terms of agonist time courses in the WT colon, the only subtle difference between ap and bl signaling was seen with the TGR5 agonists (Supplemental Fig. 2D). Ap Merck V and GP-A responses were slower in onset than their equivalent bl time courses (significantly so between 2 and 4 minutes after Merck V addition). Ap GP-A efficacy was also slower to peak and slightly blunted compared with the bl agonism, although this was not statistically significant. TERs did not change during any agonist response (Supplemental Fig. 2), indicating maintained epithelial barrier function throughout experimentation.

### GPR119 signaling in the human colon is similar pharmacologically to that in the WT mouse colon

**Figure 5.**
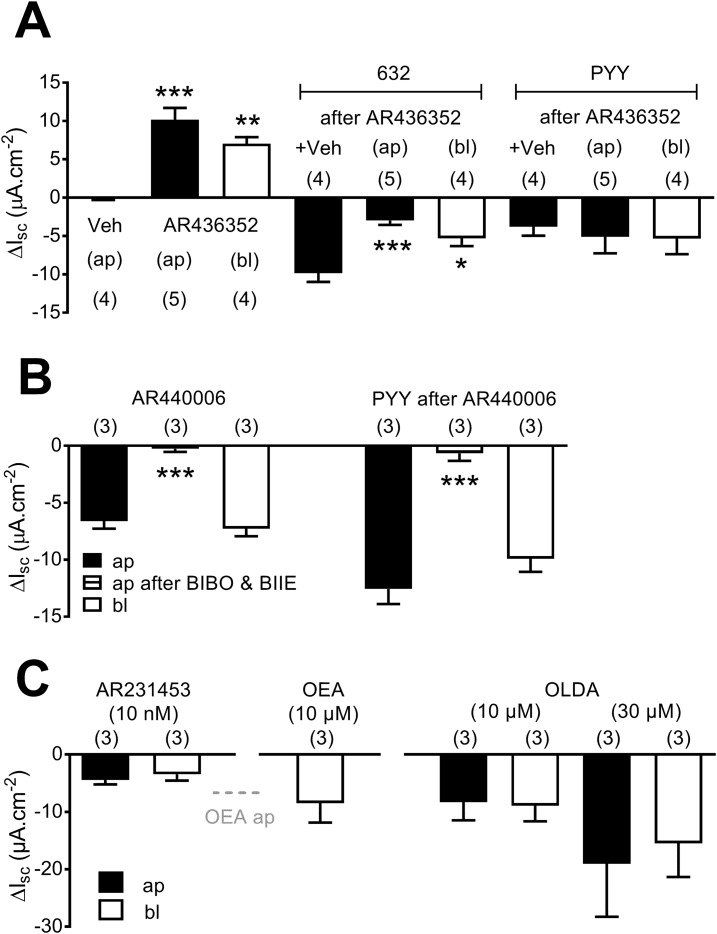
GPR119 tone and pharmacology in human colonic mucosa. (A) GPR119 antagonism (1 µM, AR436352), added to either ap or bl surfaces, is compared with vehicle (0.1% dimethyl sulfoxide) and subsequent GPR119 agonism [10 µM PSN632408 (632)], followed by PYY (100 nM) responses after either ap or bl AR436352. (B) AR440006 (3 µM) responses are similar when added to ap or bl reservoirs, and ap responses are blocked by Y receptor antagonists [300 nM BIBO3304 and 1 µM BIIE0246 (after BIBO & BIIE)]. Subsequent PYY responses (100 nM, bl) are also abolished after Y antagonist addition. (C) A comparison of ap and bl responses to AR231453, OEA, or OLDA, and the dashed, horizontal, gray “OEA ap” line denotes maximal responses to OEA (10 µM, ap), published in Cox *et al.* (8). Each bar is the means ± 1 SEM from n numbers, as shown. Statistical differences (ANOVA with Dunnett *post hoc* test) from controls are identified as follows: *P < 0.05, **P < 0.01, and ***P < 0.001.

In normal human colon mucosa, the GPR119 antagonist, AR436352, blocked tonic activity (*i.e.*, it raised basal Isc levels when added either apically or basolaterally), as seen in WT mouse tissues, and it significantly inhibited subsequent PSN632408 responses but had no affect on PYY responses (Fig. 5A). As seen in mouse tissue, AR440006 responses were a similar size, irrespective of the side on which this GPR119 agonist was added (Fig. 5B), indicating that GPR119 is located on ap and bl membranes in human colon mucosa. Notably, AR440006 responses in the human colon are PYY mediated, and the combination of Y1 and Y2 antagonists abolished the GPR119 agonism and subsequent PYY controls (Fig. 5B). Finally, AR231453, OEA, or OLDA each reduced Isc levels to a similar degree after ap or bl addition [Fig. 5C; for ap OEA responses, see (8)], again consistent with the sensitivities observed in mouse colon mucosa.

### The effects of PYY or GPR119 knockout on colonic motility and the GPR119 agonism *in vitro* and *in vivo*

**Figure 6.**
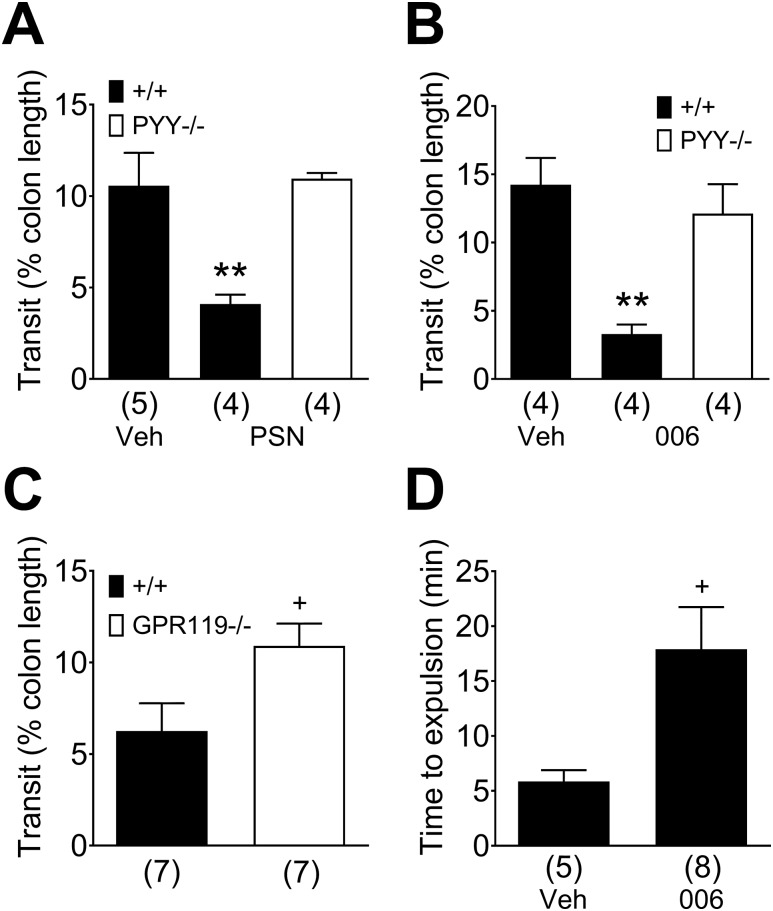
Transit is reduced by the GPR119 agonism in the WT mouse colon *in vitro* and *in vivo*. (A) Incubation of the isolated colon with PSN632408 (PSN; 10 µM) but not vehicle (Veh) slowed fecal pellet propulsion in the WT tissue (+/+; black bars) but not in the PYY^−/−^ colon (white bar). (B) Colonic transit *in vitro* was retarded by AR440006 (006; 1 µM) in the WT colon, and this activity was absent in PYY^−/−^ tissue. (C) Fecal pellet propulsion was significantly increased in the GPR119^−/−^ (white bar) compared with the WT colon (black bar). (D) AR440006 (30 mg/kg, IP) significantly slowed WT colorectal transit *in vivo* compared with vehicle (100 µl saline IP). Each bar is the means + 1 SEM from the n numbers shown in parentheses. Statistical differences were identified either using unpaired Student *t* test, **^+^***P* < 0.05, or one-way ANOVA with Dunnett post-test, **P < 0.01, for drug treatment comparisons between the WT and PYY^−/−^ colon.

Having shown that the GPR119 agonism in the mouse and human colon results in endogenous PYY release and consequent Y1 receptor signaling, we next assessed the selective GPR119 agonism on mouse colonic transit *in vitro* and *in vivo*. Fecal pellet movement *in vitro* was significantly retarded by GPR119 agonism (10 µM PSN632408; Fig. 6A), but the same stimulus had no effect on transit in the PYY^−/−^ colon (Fig. 6A). The water-soluble agonist AR440006 (1 µM) also slowed transit significantly in the WT colon and lost activity in the PYY^−/−^ tissue (Fig. 6B). The basal rate of fecal pellet transit was significantly faster in the GPR119^−/−^ colon compared with WT colonic transit (Fig. 6C). *In vivo* measurement of bead excretion in WT mice, administered IP with AR440006 (30 mg/kg), revealed that the GPR119 agonist more than doubled the time taken for bead passage compared with saline controls, indicating that the drug significantly slowed WT colonic transit (Fig. 6D). The same IP dose of AR440006 also attenuated upper-GI transit significantly in WT mice. Transit of the charcoal meal was reduced from 90.5 ± 4.1% (n = 5) after vehicle to 76.8 ± 2.1% (n = 5; *P* < 0.05) of the total small intestinal length after treatment with AR440006.

## Discussion

We have shown that a selective GPR119 agonist, AR440006, or lipids (OEA and 2-OG) elicit the same PYY-mediated, antisecretory response, irrespective of the mucosal surface to which the agonists are added. Being water-soluble, traces of AR440006 only traverse epithelial tight junctions (as determined by LC-MS/MS), and the similar kinetics of this synthetic agonist’s ap and bl responses also indicate that GPR119 is located on both L-cell domains. Importantly, this bidirectional GPR119 signaling capacity was evident in human mucosa, and these results raise the distinct advantage of luminal (or apically)-directed GPR119 activation causing endogenous PYY and incretin release. Restricting drug distribution should lower the risk of GPR119 mechanism-based side-effects.

The structure-activity comparison showed AR435707 and AR440006 to be full agonists that were less potent than the prototypic GPR119 agonist, AR231453 (2, 11, 38). The efficacy and potency of each agonist were similar for ap or bl application, indicating a similar GPR119 signaling capacity in each membrane domain. The lipids 2-OG and OEA also appeared to be full agonists at either surface, and they were slightly less potent than OLDA. Notably, all of the agonists tested, with the exception of OLDA, were sensitive to the GPR119 antagonist AR436352 (36), and their activities were absent in GPR119^−/−^ tissue (where OLDA’s was unchanged), indicating GPR119 as the primary target for all agonists but not OLDA. In addition, all agonism bar OLDAs were sensitive to Y1 and Y2 antagonists, indicating that all responses (except OLDA’s) were PYY mediated. OLDA also lacked activity at TRPV1 (35), and we infer that in the mouse colon [an area lacking a correlation between GPR119 and dopamine synthesis (35)] OLDA acts on neither GPR119 nor TRPV1. The GPR119 antagonist, AR436352, also blocked PSN632408 responses on either surface in human colon mucosa, showing further, close similarity with the GPR119 pharmacology obtained in mouse mucosae.

Basal Isc levels in WT-ascending colon mucosa were expectedly higher than those in other GI areas, given the higher Na^+^ absorptive capacity of this proximal colonic region (39). This pattern was replicated in GPR119^−/−^ tissues, with elevated Isc levels in GPR119^−/−^ jejunum and descending colon mucosae, possibly as a result of loss of substantial GPR119 inhibitory tone in these regions. The other notable difference was in GPR119^−/−^-ascending and -descending colon TER that were slightly higher than in WT mucosae, indicating raised epithelial resistance, but of unknown origin. Otherwise, the mucosae from these two genotypes were similar, revealing normal epithelial barrier function, and they were comparable with the parameters published previously for mucosae from the same range of GI areas (31, 40).

Previously, the GPR119 agonism has been shown to improve oral glucose tolerance in lean and diabetic rodents, and that glucose induces serosal secretion of intestinal PYY, GLP-1, and GIP (2, 3, 10). However, the mucosal responses that we observed in the distal colon were predominantly PYY mediated, and this is most likely because GLP-1 (and GIP) responses are relatively small in this region of the mouse colon (41) (Iain R. Tough and Helen M. Cox, unpublished GIP data). Crucially, in our 2010 study, we observed GPR119-induced GLP-1 responses in human colon mucosa but only when Y1 and Y2 receptors were blocked (8). Therefore, it is likely that GLP-1 and PYY are coreleased, and their responses are coincident upon GPR119 stimulation in human mucosae, but the lower levels of GLP-1 receptor expression in the mouse colon limit GLP-1 signaling compared with the more robust PYY-mediated Y1 epithelial mechanism in this tissue (22, 23, 31, 40). Differential PYY and GLP-1 release is, however, also possible, given that the peptides can be packaged in different vesicle populations within L-cells (42), and this may also explain why certain ligands (*e.g.*, for FFA2) appear to act solely via PYY (34).

To minimize GPR119 desensitization in mucosae, we added one agonist concentration per preparation and monitored non-normalized responses for 20 to 30 minutes. Therefore, we were surprised to find that the most potent ligand, AR231453, was a partial agonist compared with the slightly less potent agonists AR435707 and AR440006 and the lipids OEA and 2-OG in mouse-descending colon mucosa. In a previous study, also using single additions, the potent agonist PSN-GPR119 also exhibited partial efficacy (∼50%) (10) compared with PSN632408 (8) and other agonists tested in the current study. Why AR231453 is partially active in native mouse tissue when it apparently exhibits full agonism via G*α*s signaling in isolated murine L-cells and when it binds apparently to similar epitopes on the GPR119 receptor as OEA (17, 38) have yet to be resolved. Nevertheless, this agonist exhibited efficacy *in vivo*, reducing gastric emptying (28), improving glucose tolerance in WT mice and in *β*-cell-specific GPR119 knockouts (29), and showing the functional significance of GPR119 incretin signaling in glucoregulation. Importantly, Moss *et al.* (27) showed that L-cell knockout of GPR119 abolished elevations in plasma GLP-1 after oral gavage of an olive and corn-oil mixture. Acute fat exposure stimulated GLP-1 secretion in lean humans (as well as increasing duodenal GPR119 but not FFA1 or FFA4 expression), whereas habitual consumption of polyunsaturated fatty acids was negatively correlated with GPR119 expression (43). Thus, GPR119 appears to be an early transcriptional responder to duodenal fat, and our own studies show clear PYY responses to acute luminal GPR119 agonists in human colonic mucosa; thus, GPR119 is a likely lipid sensor along the length of the human GI tract.

GPR119 is constitutively active (17), especially when signaling via G*α*s in recombinant cells (18) and in native L-cells (27). Constitutive GPR119 activity may contribute to the endogenous GPR119 tone that we observed in the mouse and human colon using the GPR119 antagonist AR436352 (36). It is possible that locally produced lipids, such as OEA, provide a degree of endogenous ligand-activated GPR119 activity (24, 44) and subsequent PYY release. With the use of an FAAH blocker at a concentration shown to elevate endogenous OEA levels and to amplify GPR119 signaling (25), we revealed enhanced GPR119 and Y1 tonic activities in WT but not in PYY^−/−^ tissues following URB597 pretreatment. It is notable that the blocking of MC4, another L-cell-expressed receptor (with the antagonist HS-014) (30) revealed tonic MC4 activity that was unaffected by ablation of GPR119 or by FAAH inhibition. We conclude that endogenous OEA may contribute to GPR119 and Y1 tone, but it is not apparently required for MC4 tone [for which the endogenous mediator(s) remain to be identified]. Y1 and Y2 tones were, however, present in the GPR119^−/−^ colon, showing signaling downstream of L-cell activation; *i.e.*, PYY responses were normal in the GPR119^−/−^ tissue. Taken together, we conclude that several endogenous mechanisms—GPR119 being one important pathway—stimulate L-cells and PYY responses within mucosae and notably, that this mechanism extends to retarding colonic and upper-GI motility.

The activities of agonists specific for other GPCRs known to be highly expressing on L-cells highlight the breadth of signaling, with functional relevance in enteroendocrine cells (23, 37). Selective FFA1 and FFA4 mucosal signaling (albeit at single agonist concentrations) was similar in the WT and GPR119^−/−^ colon, and their response time courses after ap or bl addition were coincident. These LCFA-activatable pathways are also PYY, Y1 mediated and glucose sensitive (16), as is the FFA2 agonism (34). The selective FFA3 agonism was also unchanged in the GPR119^−/−^ colon, but this short-chain fatty acid sensor is markedly different from FFA2 and other nutrient-sensing mechanisms discussed thus far. Intestinal FFA3 signaling does not appear to be L-cell or PYY/GLP-1 mediated. Instead, the FFA3 agonism (with AR420626) involves enteric neurons (45) and is glucose independent in mouse colonic mucosa (Iain R. Tough and Helen M. Cox, unpublished data), and this mechanism was also unaltered in the GPR119^−/−^ colon. Acute short-chain fatty acid activation of FFA2 and FFA3 leads to coincident L-cell and submucosal enteric neuron activation (Iain R. Tough and Helen M. Cox, unpublished data). Interestingly, commensal microbes can also generate *N*-acyl amides with affinity for GPR119, so in addition to its fat-sensing function, luminally directed GPR119 may sense certain microbial metabolites (46). Finally, we found that acute ap TGR5 responses with Merck V were comparable in the WT and GPR119^−/−^ colon, but these responses exhibited a 2-minute latency compared with the faster bl Merck V responses. A similar latency was observed with ap administration of another TGR5 agonist, GP-A [at the same concentration used by Brighton *et al.* (33)], which exhibited a bl preference measuring GLP-1 release from ileal mucosa, over a 60-minute period. It is possible that these TGR5 agonists cross GI mucosae to activate predominant basolaterally located TGR5, but the peak epithelial responses that we observed were not significantly different within 10 minutes, although ap GP-A responses were blunted. Potentially, the sidedness of the TGR5 agonism may resolve further over 1 hour or longer time periods, as observed *in vivo* for peptide release following systemic, rather than luminal, TGR5 agonist exposure (47). Nevertheless, in our study, the acute L-cell signaling initiated by the ap TGR5 agonism was consistent in WT and GPR119^−/−^ colonic mucosa, as was FFA1, FFA3, and FFA4 signaling.

In summary, our mucosal studies have found that ap as well as bl GPR119 agonism have the potential to stimulate L-cell PYY release with paracrine consequences that extend to the slowing of upper-GI and colonic motility. We propose that this glucose-dependent L-cell response to a gut-restricted GPR119 stimulus has therapeutic potential, either alone or in combination with peptide-stabilizing drugs, in modulating insulinotropic signaling with reduced risk of hypoglycemia.

## Supplementary Material

Supplemental Data

## Abbreviations:

2-OG: 2-monoacylglycerol
ANOVA: analysis of variance
ap: apical
bl: basolateral
EC_50_: half-maximal effective concentration
FAAH: fatty acid amide hydrolase
FFA: free fatty acid
FFA1: free fatty acid receptor 1
FFA3: free fatty acid receptor 3
FFA4: free fatty acid receptor 4
GI: gastrointestinal
GIP: glucose-dependent insulinotropic peptide
GLP-1: glucagonlike peptide 1
GPCR: G protein–coupled receptor
GPR119: G protein–coupled receptor 119
GPR119^−/−^: G protein–coupled receptor 119 deficient
IP: intraperitoneal
Isc: short-circuit current
KH: Krebs-Henseleit
LC: liquid chromatography
LCFA: long-chain free fatty acid
*m*/*z*: mass-to-charge ratio
MC4: melanocortin 4 receptor
MS: mass spectrometry
MS/MS: tandem mass spectrometry
OEA: oleoylethanolamide
OLDA: *N*-oleoyldopamine
PYY: peptide YY
PYY^−/−^: peptide YY deficient
SEM: standard error of the mean
SGLT1: sodium/glucose cotransporter 1
t: time
TER: transepithelial resistance
TRPV1: transient receptor potential vanilloid 1
VIP: vasoactive intestinal polypeptide
WT: wild-type
*α*-MSH: *α*-melanocyte–stimulating hormone
