# Supplementary material for: Bidirectional GPR119 Agonism Requires Peptide YY and Glucose for Activity in Mouse and Human Colon Mucosa
Source: Endocrinology. 2018 Feb 19;159(4):1704–17. doi: 10.1210/en.2017-03172 (PMC5972582; doi:10.1210/en.2017-03172)
Supplement: Supplemental Data [file en.2017-03172.sd1.pdf]

SUPPLEMENTARY DATA

Genotype		Jejunum	Terminal Ileum	Ascending Colon	Descending Colon
WT	I_{sc} ($\mu A.cm^{-2}$)	37.2 ± 5.6 (*n*=8) ++	35.2 ± 6.9 (*n*=8) ++	71.0 ± 4.7 (*n*=8)	45.3 ± 2.4 (*n*=80) ++
	TER ($\Omega.cm^2$)	23.9 ± 2.0 (*n*=8)	17.8 ± 2.5 (*n*=8)	23.6 ± 1.6 (*n*=8)	27.6 ± 1.5 (*n*=80)
GPR119-/-	I_{sc} ($\mu A.cm^{-2}$)	57.8 ± 5.8 (*n*=8) *	51.4 ± 5.8 (*n*=8)	64.0 ± 8.0 (*n*=8)	53.2 ± 2.8 (*n*=88) *
	TER ($\Omega.cm^2$)	25.9 ± 3.4 (*n*=8)	20.6 ± 4.2 (*n*=8)	36.9 ± 4.1 (*n*=8) *	32.4 ± 1.6 (*n*=88) *

Supplementary Table 1. Electrophysiological parameters (Isc and TER) for different intestinal tissues from WT and GPR119-/- mice. Values are the mean ± SEM with statistical differences as shown. $^{++}$ *P* < 0.01 compared with WT ascending colon (one-way ANOVA with Tukey's post-test). * *P* < 0.05 compared with WT values in the same GI area (Student's unpaired t-test).

Supplementary Figure 1

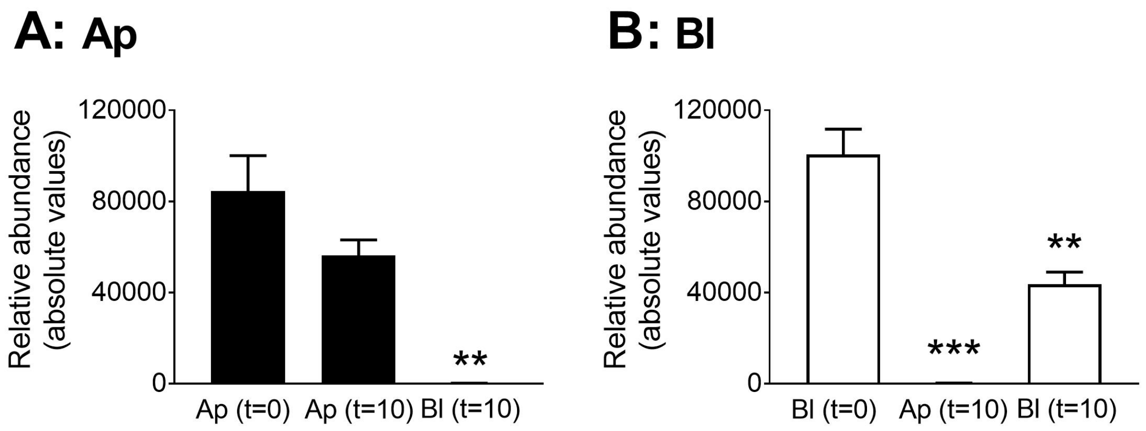

Supplementary Figure 1. LC-MS/MS analysis of apical and basolateral AR440006. Assays were started with additiona of AR440006 (1 μM; t = 0 min) to either the apical (Ap) or basolateral (Bl) reservoir. After 10 min samples were taken from both Ap and Bl reservoirs, diluted in 0.1% formic acid in 50% methanol and analysed by LC-MS/MS. Data from 3 different colonic specimens (mean ± 1 SEM) shows the relative abundance of AR440006 (calculated from the area of peaks with retention time of 4.2 min and product ion m/z 280) compared with initial levels of AR440006 at t = 0 min, using ANOVA with Dunnett's post-test (** $P < 0.01$, *** $P < 0.001$).

Supplementary Figure 2.

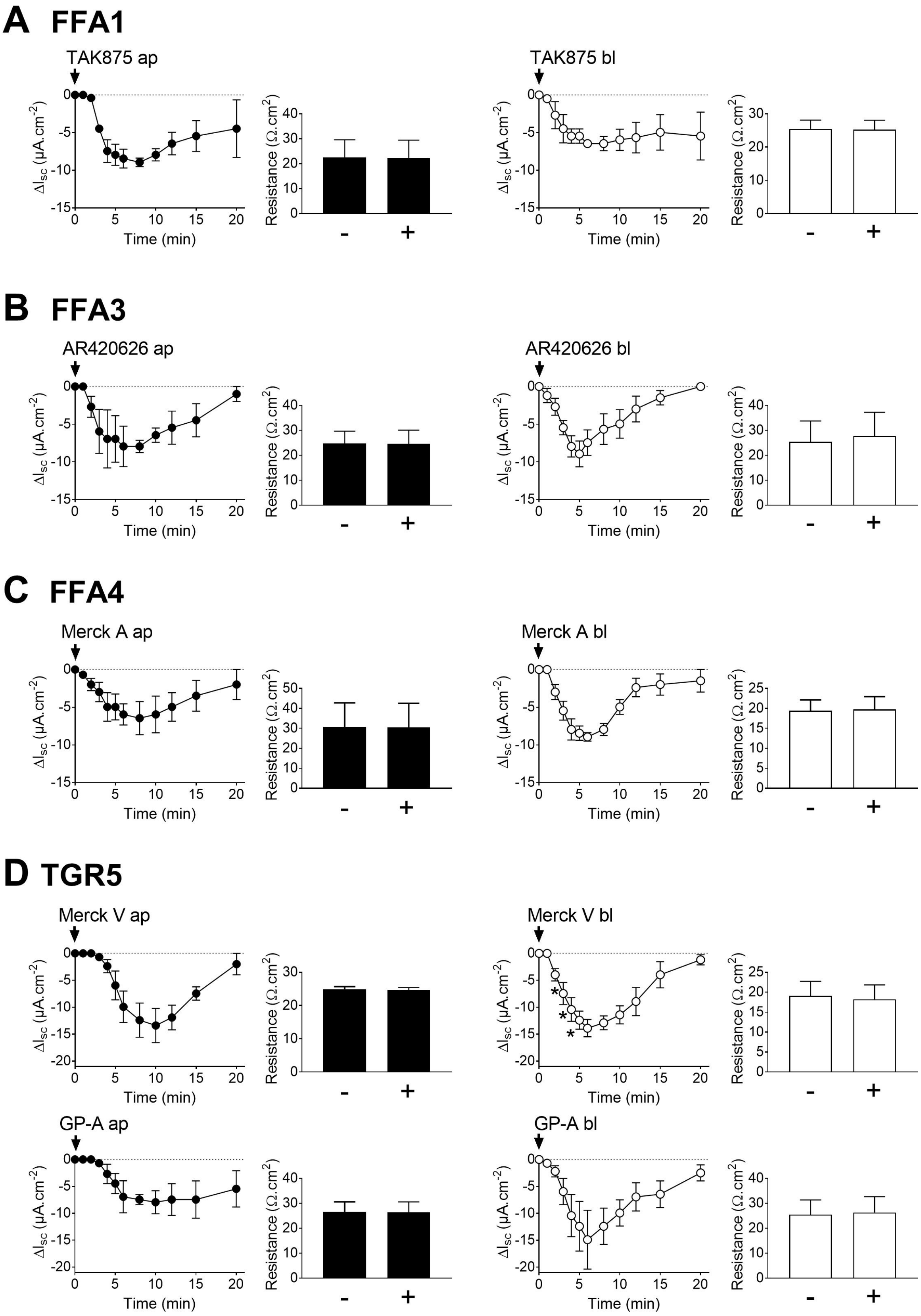

Supplementary Figure 2. Agonist time-courses with accomanying TER measurements in WT mouse colon mucosa after either apical (ap, black symbols) or basolateral (bl, white symbols) drug addition at t = 0 min (' - ') and at peak response (t = 5 - 10 min; ' + '). Agonists were TAK875 (3 μM), AR420626 (100 nM), Merck A (10 μM), Merck V (1 μM) and, the TGR5 agonist GP-A (3 μM) as used by Brighton *et al*. (33). Data are the means ± 1 SEM (*n* = 4 throughout) and statistical differences between basolateral and apical time-points for the same agonist (see Merck V) or between TER values, were determined using Student's unpaired *t*-test (* $P < 0.05$).
